# An Animal Model for the Juvenile Non-Alcoholic Fatty Liver Disease and Non-Alcoholic Steatohepatitis

**DOI:** 10.1371/journal.pone.0158817

**Published:** 2016-07-08

**Authors:** Veronica Marin, Natalia Rosso, Matteo Dal Ben, Alan Raseni, Manuela Boschelle, Cristina Degrassi, Ivana Nemeckova, Petr Nachtigal, Claudio Avellini, Claudio Tiribelli, Silvia Gazzin

**Affiliations:** 1 Fondazione Italiana Fegato, AREA science Park, Trieste, Italy; 2 IRCCS Ospedale Burlo Garofolo Pediatric Hospital, Clinical Chemistry Laboratory, Trieste, Italy; 3 Medical Research Institute, AREA science Park, Trieste, Italy; 4 Department of Biological and Medical Sciences, Faculty of Pharmacy in Hradec Kralove, Charles University in Prague, Heyrovskeho 1203, Hradec Kralove, 500 05, Czech Republic; 5 Azienda Ospedaliero-Universitaria “Santa Maria della Misericordia”, Dip. Anatomia e Patologia, Udine, Italy; 6 Department of Medical Sciences, University of Trieste, Trieste, Italy; Bambino Gesù Children's Hospital, ITALY

## Abstract

Non Alcoholic Fatty Liver Disease (NAFLD) and Non-Alcoholic Steatohepatitis (NASH) are the hepatic manifestations of the metabolic syndrome; worrisome is the booming increase in pediatric age. To recreate the full spectrum of juvenile liver pathology and investigate the gender impact, male and female C57Bl/6 mice were fed with high fat diet plus fructose in the drinking water (HFHC) immediately after weaning (equal to 3-years old human), and disease progression followed for 16 weeks, until adults (equal to 30-years old human). 100% of subjects of both genders on HFHC diet developed steatosis in 4weeks, and some degree of fibrosis in 8weeks, with the 86% of males and 15% of females presenting a stage 2 fibrosis at 16weeks. Despite a similar final liver damage both groups, a sex difference in the pathology progression was observed. Alterations in glucose homeostasis, dyslipidemia, hepatomegaly and obese phenotype were evident from the very beginning in males with an increased hepatic inflammatory activity. Conversely, such alterations were present in females only at the end of the HFHC diet (with the exception of insulin resistance and the hepatic inflammatory state). Interestingly, only females showed an altered hepatic redox state. This juvenile model appears a good platform to unravel the underlying gender dependent mechanisms in the progression from NAFLD to NASH, and to characterize novel therapeutic approaches.

## Introduction

The increasing prevalence of the metabolic syndrome and obesity even at young age (< 5 years-old) is threatening the public health system. The metabolic syndrome, referred as a complex phenotype with obesity, dyslipidemia, insulin resistance, increase in visceral adiposity, etc., is closely associated to liver complications such as non-alcoholic fatty liver disease (NAFLD) and its progression towards more severe stages such as non-alcoholic steatohepatitis (NASH), which can leads to more severe liver diseases (cirrhosis and hepatocellular carcinoma) [1]. Indeed, is expected that in the next 20 years the most frequent indication for liver transplantation will be associated to metabolic disorders rather than the current viral-related damages [2,3]. The increasing incidence of NAFLD/NASH is a mirror of the parallel spread of hypercaloric diet, associated with fructose-containing drinks and sedentary life habits among children and adolescents. The metabolic and hormonal aspects of a growing subject [1,4,5] have been indicated as possible factors influencing the progression of the disease [6–9]. Although precise estimation of incidence rates are uncertain, sex differences seem to be present. It is well accepted that males are more predisposed to develop NAFLD, with a burst of hepatic injury [10,11] and a rapid accumulation of visceral fat [12]. An homologous situation has been reported in women only after menopause as a consequence of estrogen decline [13].

Generally speaking, NAFLD onset is characterized by an abnormal fat accumulation within the liver (simple steatosis) which can be reversed by life-style changes. The progression from simple steatosis to NASH (characterized by cellular inflammation, ballooning degeneration and fibrosis) is determined by the initiation of a fibrotic response which enhances the risk to further progress towards more severe liver injuries (such as cirrhosis and hepatocellular carcinoma) [1]. Nevertheless, there are still large gaps in the knowledge of the pathogenesis, progression, diagnosis and treatment of these conditions. Thus, there is an urgent need to fill in these gaps, and animal models offer the possibility to study the mechanisms that so far remained unrevealed.

The search for factual NAFLD/NASH animal models, able to recreate the causative, morphological, biochemical and clinical features of the human pathology over the last years, is well documented (reviewed elsewhere [14–17]). Both genetic and nutritional models have been widely used. However, many of these models missed in fully reproducing the clinical condition, either because not representative of the general population (genetically modified rodents), or because the causative mechanisms differ significantly from what occurs in clinic NAFLD/NASH (deficient diets). Recently a substantial improvement has been achieved by the combination of the hypercaloric fatty diet with fructose [18–21]. Fructose addition is not only instrumental to induce hepatic fibrosis in rodent models, but most importantly mimics the increased consumption of high-fructose corn syrup in human alimentary habits [21].

Nevertheless, these nutritional models have been recreated only in adult animals (feeding starts most commonly at 8–10 weeks old), and females have been largely omitted, (except for those studies on ovariectomized sexually mature mice, used to assess the supposed liver protective effect of estrogens [1,22–24]).

Given the data previously reported *in vivo* adult models and their limitations, the aim of the present study was to develop and characterize the first consistent juvenile animal model able to reproduce the context in which human NASH develops and to explore all the described clinical events involved in the progression from NAFLD to NASH.

For this purpose, C57Bl/6 mice were exposed to high-fat diet supplemented with fructose in drinking water (HFHC, where C = carbohydrates). Diets (control and HFHC) were started immediately after weaning (3weeks old mice, comparable to ~ 3 years old human), and continued for 16weeks (when mice were 19weeks old, ~ 30years old human). Challenge with HFHC diet started when animals were still sexually undifferentiated (pups) and continued until the adult age. Both males and females were considered as separated groups in order to explore gender differences in the development of NAFLD and progression to NASH during the adolescence. The pathology evolution was screened every 4 weeks conducting the studies currently used in daily clinical practice (anthropometric and biochemical parameters, histopathological analysis).

## Materials and Methods

### Experimental set-up: Animals and diet

C57Bl/6 mice pups (37 of each sex) were obtained from Harlan Laboratories S.R.L. (S.Piero al Natisone, Italy). Immediately after weaning, animals were randomly group-housed in cages in a temperature-controlled environment (22±2°C) and on a 12 hours light/dark schedule, and fed *ad-libitum* with control diet (CTRL, D12328, *Research Diets*, New Brunswick, NJ) or HFHC diet (HFHC: D12331, *Research Diets*, New Brunswick, NJ), plus 42g/L fructose/sucrose in drinking water, for a total of 16 weeks [25]. Four experimental checkpoints were established (4, 8, 12 and 16 weeks of diet), in which part of the animals was sacrificed. Animal care and procedures were conducted in accordance with the Italian Law (decree 116–92) and by European Community directive 86-609-EEC. All experimental protocol were approved by the local committee of the Medical Research Institute and by the National Authority (Ministero della Salute—Direzione generale della sanità animale e dei farmaci veterinari—Ufficio VI, Rome, Italy, under the Article 7, D.lgs 116/92). The maximal effort was done to reduce the number of animals used and their sufferance in the respect of the 3R rule.

### Sample collection at the experimental checkpoints

Liver, aorta, adipose tissue and blood samples were collected at each experimental checkpoint time. After O/N fasting, animals of both sexes and diet groups (4, 8,12 weeks n = 3–5 mice *per* group; 16 weeks n = 5–8 mice *per* group) were deeply anesthetized (Zoletil 10mg/kg and Xilazine 5mg/kg of body weight, intra-peritoneal). Blood was collected by cardiac puncture (exsanguination). Whole blood was allowed to clot at room temperature (RT) for 20 minutes, followed by centrifugation at 3500g for 5 minutes at RT to separate the serum.

### Body weight, liver weight, epididymal adipose tissue weight

Body weight was recorded weekly, while liver and epididymal fat pads tissues were dissected and weighted only at each experimental checkpoint time. Moreover, sacrificed animals’ body weight and naso-anal length were recorded for indirect computation of body composition via body mass index (BMI), for the confirmation of the development of the obese phenotype [26].

### Serum triglyceride, cholesterol, low and high-density lipoprotein quantification

Serum triglycerides (TG), total cholesterol (Chol), high-density lipoprotein cholesterol (HDL-C) and low density lipoprotein cholesterol (LDL-C) contents were assessed at every experimental checkpoint time. Enzymatic colorimetric kits (Roche Diagnostics GmbH; Mannheim, Germany) were used to measure these parameters in a Roche HITACHI Cobas e501 instrument (Roche Diagnostics GmbH; Mannheim, Germany) accordingly to manufacturer’s instructions.

### Assessment of glucose homeostasis, serum insulin and insulin resistance

Glycaemia and insulinemia were measured after 6-hours fasting every four weeks (two days before sacrifice), collecting few blood drops from submandibular vein (about 40μL *per* animal) in local lidocaine anesthesia. Glucose was measured in whole blood using One Touch verio IQ^®^ meter (Life Scan Europe, Zug, Switzerland) accordingly to manufacturer’s instructions. Serum insulin content was quantified from the same blood sample, by AlphaLISA Insulin Kit (Perkin Elmer, Waltham, USA) following manufacturer’s instructions. The homeostasis model assessment of insulin resistance HOMA-IR was calculated for each animal according to the following formula: blood glucose (mg/dL) x fasting insulin (μU/mL)/405 [27].

### Quantification of hepatic aminotransferases

As a measurement of liver damage, serum alanine (ALT) activity was quantified at each checkpoint by enzymatic colorimetric assay (Roche Diagnostics GmbH; Mannheim, Germany) accordingly to manufacturer’s instructions in a Roche HITACHI Cobas e501; (Roche Diagnostics GmbH; Mannheim, Germany).

### Histopathological analysis

At each checkpoint, the liver was harvested and a portion of tissue immediately fixed in buffered formalin (formaldehyde 4%, NaH2PO4 4gr/L, Na2HPO4 6.5 gr/L: pH 6.8). Tissue sections were cut at a thickness of 3.5 μm and stained with Hematoxylin & Eosin (H&E) and Gömöri trichrome stain. Histology was read by a single independent pathologist, blinded to experimental design and treatment groups. Scores were attributed to steatosis (0–3), lobular inflammation (0–3), portal inflammation (0–1), and ballooning (0–2). Fibrosis was analyzed separately with a scale from 0 to 4 [28,29]. Likewise, aortae were also collected at each checkpoint to assess the atheroma plaque formation. Isolated aorta was immersed in OCT (Optimal Cutting Temperature) embedding medium (Leica, Czech Republic) and frozen at -80°C. Serial sections (7μm thick) were cut on a cryostat and placed on gelatin-coated slides. Sections were stained with Hematoxylin and Eosin (H&E). Photomicrographs and image digitizing were performed using Olympus AX 70 (Olympus, Czech Republic) with a digital camera Pixelink PL-A642 (Vitana Corporation, Canada).

### Gene Expression analysis by Real Time q-PCR

cDNA samples obtained from RT-PCR were used in the Real Time quantitative PCR to determine α-SMA gene expression. PCR amplification was carried out in 25μL reaction volume containing cDNA, 1x iQ SYBR Green Supermix (Bio-Rad Laboratories, Hercules, USA) and specific sense and anti-sense primers: α-SMA (forward GGCATCAATCACTTCAAC; reverse TCTGGTCACCTGTATGTA) and Col1A1 (forward AAGAAGACATCCCTGAAG; reverse ATACAGATCAAGCATACCT). Primer pairs used for Real Time PCR were designed using the software Beacon Designer 8.10 and they were synthesized by Sigma Genosys Ltd.

In order to verify the specificity of the amplification, a melt-curve analysis was performed, immediately after the amplification protocol. Non-specific products of PCR were not found. The relative quantification was made using the Pfaffl modification of the ΔΔCt equation [30], taking into account the efficiencies of the gene. The results were normalized to GAPDH (reverse: GCAGAAGGGGCGGAGATGAT, forward: GCAGTGGCAAAGTGGAGATT) and β-actin (reverse: AAGCCTTCATACATCAAGTTG forward: GTTGGTTGGAGCAAACATC) used as reference genes. The initial amount of the template of each sample was determined as relative expression *versus* the control sample, which is considered the 1 x sample. All Real Time PCR reactions were performed with the following temperature protocol: Cycle 1 (1X) 30 sec at 95°C, Cycle 2 (40X) 5 sec at 95°C and 20 sec at 60°C, Cycle 3 (1X) 60 sec at 95°C, Cycle 4: (1X) 60 sec at 55°C, Cycle 5: (80X) 10 sec at 55°C-94.5°C. Real Time PCR was performed using the iQ5™ Multicolor Real-Time Detection System (Bio-Rad Laboratories, Hercules, USA). The data were analyzed using iQ5™ optical system software version 2.0 (Bio-Rad Laboratories, Hercules, USA).

### Collagen determination after Sirius Red staining

The collagen content of the liver was assessed by Lopez-De Leon and Rojkind colorimetric method, using Sirius Red for the staining of collagenous proteins and Fast-Green for non-collagenous proteins. At each checkpoint, the liver was harvested and a portion of tissue immediately fixed in buffered formalin (formaldehyde 4%, NaH2PO4 4gr/L, Na2HPO4 6.5 gr/L: pH 6.8). Tissue sections were cut at a thickness of 3.5 μm and layered on glass slides. Slices were deparaffinized after incubation with xylenes (2x5 min), ethanol 100% (2x 2min), ethanol 95% (2x 2min), ethanol 70% (1x 2min), ethanol 50% (1x 2min) and distilled water (2x2min). The slices were stained with picro-sirius red stain solution (0.1% direct red 80, 0.1% fast green FCF dissolve in 1.2% aqueous picric acid solution) and incubated for 60min. After the staining, the slides were rapidly dipped in acidified water to destain and finally quickly dehydrate and mount in xylene. Absorbance of stained Sirius red collagen was quantified after dye elution with 1 mL of 0.1% NaOH in absolute methanol (1:1). The eluted color was read immediately in a Beckman DU 640B spectrophotometer at 540 and 605 nm, the maximal absorbance of Sirius red and Fast green FCF respectively. In order to calculate the concentration of collagen we followed the procedure reported by Lopez-De Leon and Rojkind [31].

### Oxidative stress determination by MDA quantification

Oxidative stress was assessed measuring MDA, one of the aldehydic lipidperoxidation products through the generation of colored products, after the reaction with thiobarbituric acid (TBA). Hepatic tissue was cut by medical scissors and homogenized on ice, using 10mM Sodium Phosphate, 1mM EDTA, 0.2 mM BHT buffer. The homogenate was centrifuged at 10,000 g, 4°C for 10 minutes and supernatant was collected for MDA detection. Briefly, each sample was prepared by adding TBA (0.7% w/v) BHT (2% w/v in ethanol) SDS 8.1% and acetic acid 10% v/v. After 45minutes heat at 100°C pink pigments were formed. After a quickly cooling process, the samples were centrifuged at 10,000 g for 5 minutes. The supernatant was collected and added to a same volume of TCA 10%, in order to precipitate the proteins. The solution was vortexed and centrifuged at 15,000 g for 3 minutes. The supernatant was collected for absorbance detection at 440nm, 532nm and 600nm. MDA was calculated using Hodges et al. equation [32].

### Statistical analysis

If not differently stated, data are reported as mean ± SD of 3–5 (4, 8, 12 weeks) or 5–8 (16 weeks) different animals of each experimental group *vs*. sex and age-matched control. Statistical comparison between diet groups was performed using one way ANOVA and post-hoc Tukey-Kramer’s test. A p-value of <0.05 was considered statistically significant.

## Results

### Body, liver and epididymal adipose tissue weight

**Fig 1:**
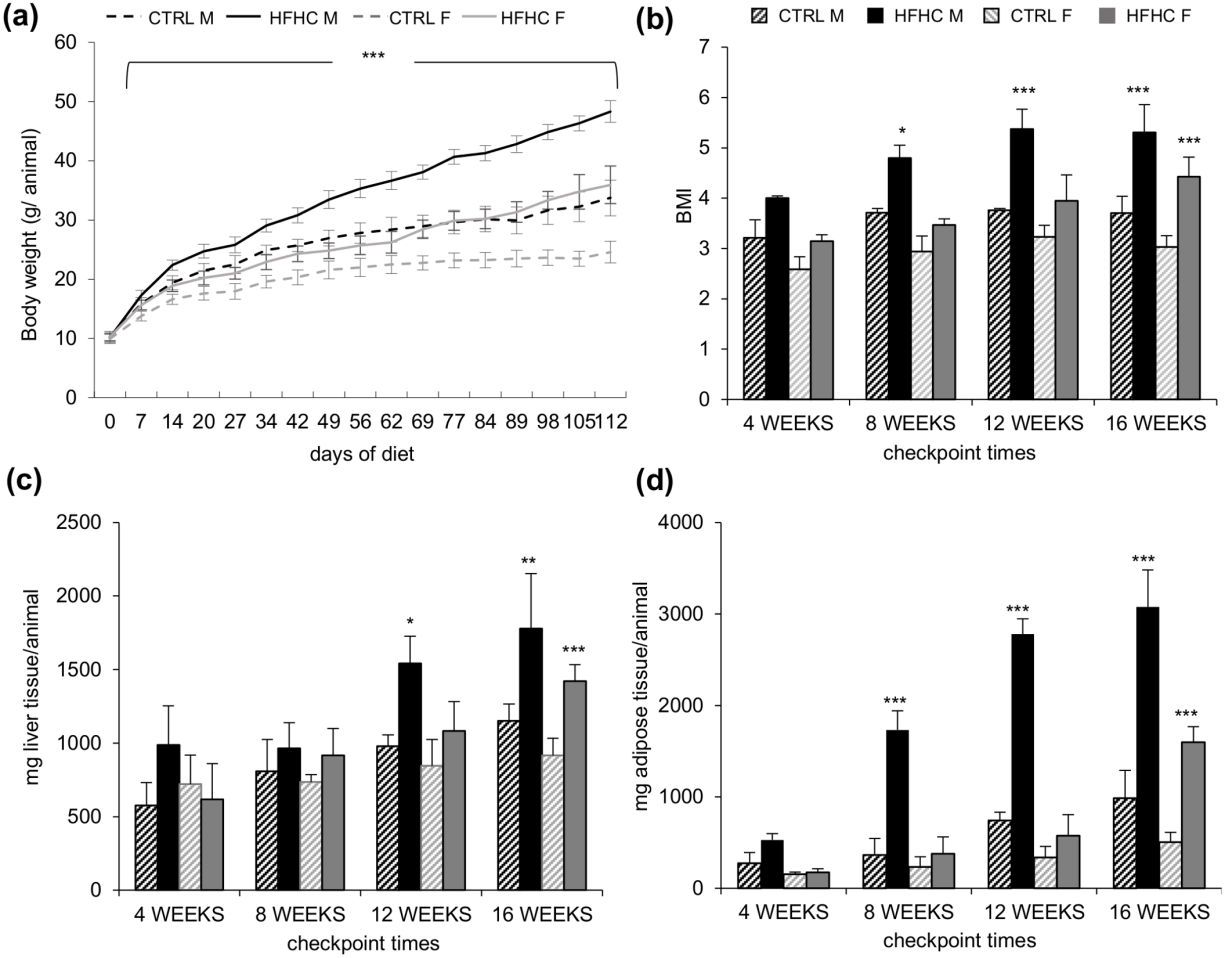
Representation of the macroscopic parameters. **(a)** Weekly body weight increase during the experimental time course. **(b)** BMI. **(c)** Liver weight. (**d)** Epididymal fat pads weight. Data are expressed as mean ± S.D. Statistical significance was calculated *vs*. age and sex matched CTRL. * p<0.05, *** p<0.001.

HFHC diet induced a significant increment in body weight from the first week both in males and females (p<0.001) as compared to sex and age-matched control animals (Fig 1a). At the end of the experimentation period (week 16) the weight of HFHC males was 55.91±3.17 gr/animal, significantly higher than in controls (33.72±3.04 gr/animal; p<0.001). The extent of increment in females was lower (48.3±5.0 gr/animal in the HFHC group *vs*. 24.58±1.82 gr/animal in control diet, p<0.001).

The body mass index (BMI) leaded to a marked overweight (ratio ≥ 1.25, based on human data) from week 4 in males (p = 0.06), and an obese phenotype (ratio ≥ 1.39) at week 12 (p<0.001) (Fig 1b). On the other hand, HFHC females showed signs of overweight only after week 12 and obesity at week 16 (p<0.001).

Liver weight also increased progressively, becoming significant after 12 weeks in males (1.6±0.2 folds, p<0.05) and at week 16 in females (1.5±0.1 fold, p<0.001) (Fig 1c).

The hypertrophy of epididymal fat, as an indirect measurement of central adiposity, showed a sustained and progressive increase in males with a significant difference starting at week 8 (4.7±0.6 folds, p<0.001). As for the liver weight, the difference was evident only after 16 weeks (3.2±0.3 folds, p<0.001) (Fig 1d) in females.

### Assessment of glucose homeostasis, serum insulin and insulin resistance

**Fig 2:**
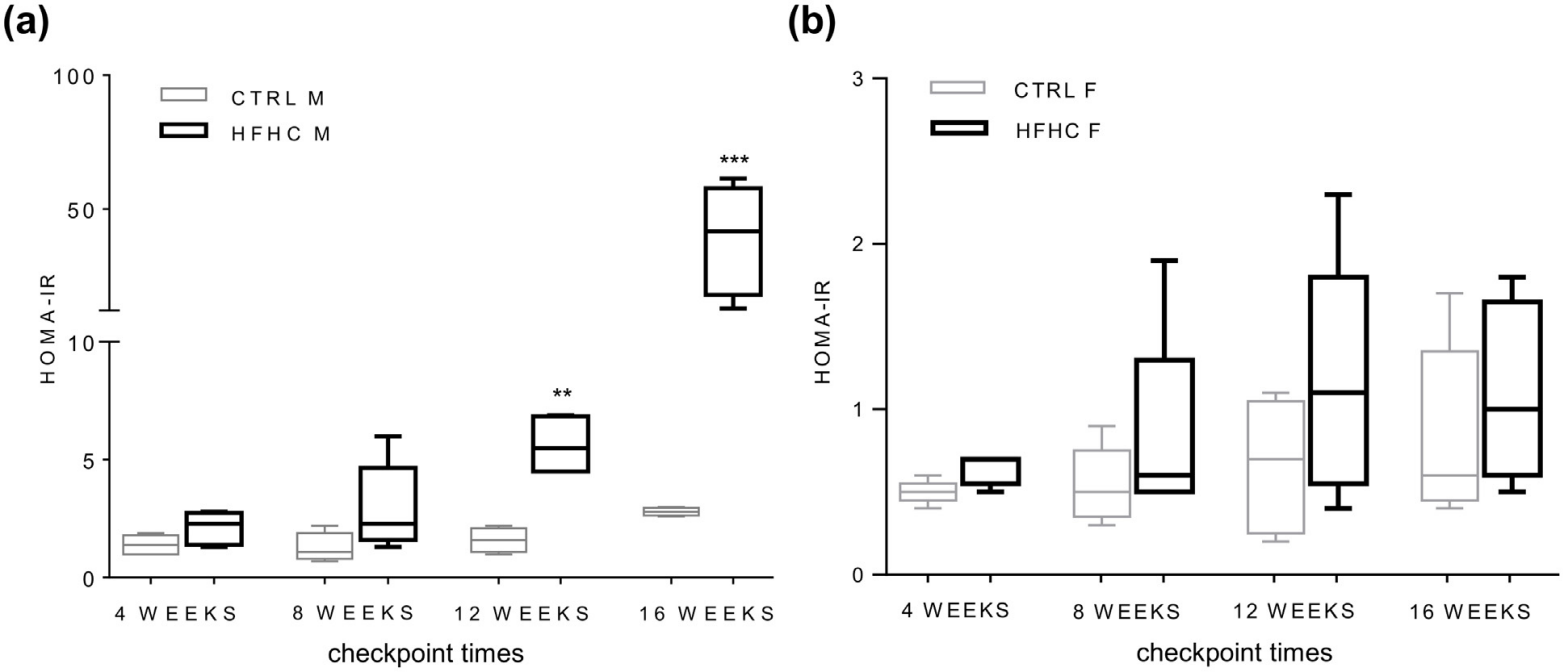
HOMA-IR index box plot. **(a)** Results in control (CTRL) and High Fat High Carbohydrates (HFHC) males (M). **(b)** Results in control (CTRL) and High Fat High Carbohydrates (HFHC) females (F). Statistical significance was calculated *vs*. age and sex matched CTRL. ** p<0.01, *** p<0.001.

HFHC diet induced hyperglycemia in both sexes from week 12 (Table 1). Interestingly, an increase in insulinemia was observed only in males (week 12), while in females this parameter was unchanged at all the experimental checkpoints (Table 1). In line with this observation, HOMA-IR index (Fig 2a), was increased from week 12 only in HFHC males (2.8±0.5 folds, p<0.01) and even more at week 16 (13.8±7.1 folds, p<0.001); conversely females never showed insulin resistance (Fig 2b).

**Table 1:** Glucose and insulin quantification in males and females. ^a^ p<0.05, ^b^ p<0.01, ^c^ p<0.001, ns: not statistically relevant. Mean ± SD *vs*. age and sex matched CTRL.

	MALE				FEMALE			
Parameter	4 weeks	8 weeks	12 weeks	16 weeks	4 weeks	8 weeks	12 weeks	16 weeks
Glucose mg/dL	241.5±16.6	203.5± 23.8	244.0± 36.0 ^c^	279.7± 49.4 ^c^	180.7± 24.4	159.2± 30.2	193.8± 20.4 ^b^	201.6± 32.6 ^c^
Insulin μU/mL	3.6±1.3	6.0±3.5	10.3±2.2 ^b^	56.9±28.9 ^c^	1,4±0.2	1.5±0.27	2.5±1.3	2.3±0.9

### Serum cholesterol, HDL-C, LDL-C and triglyceride quantification

**Fig 3:**
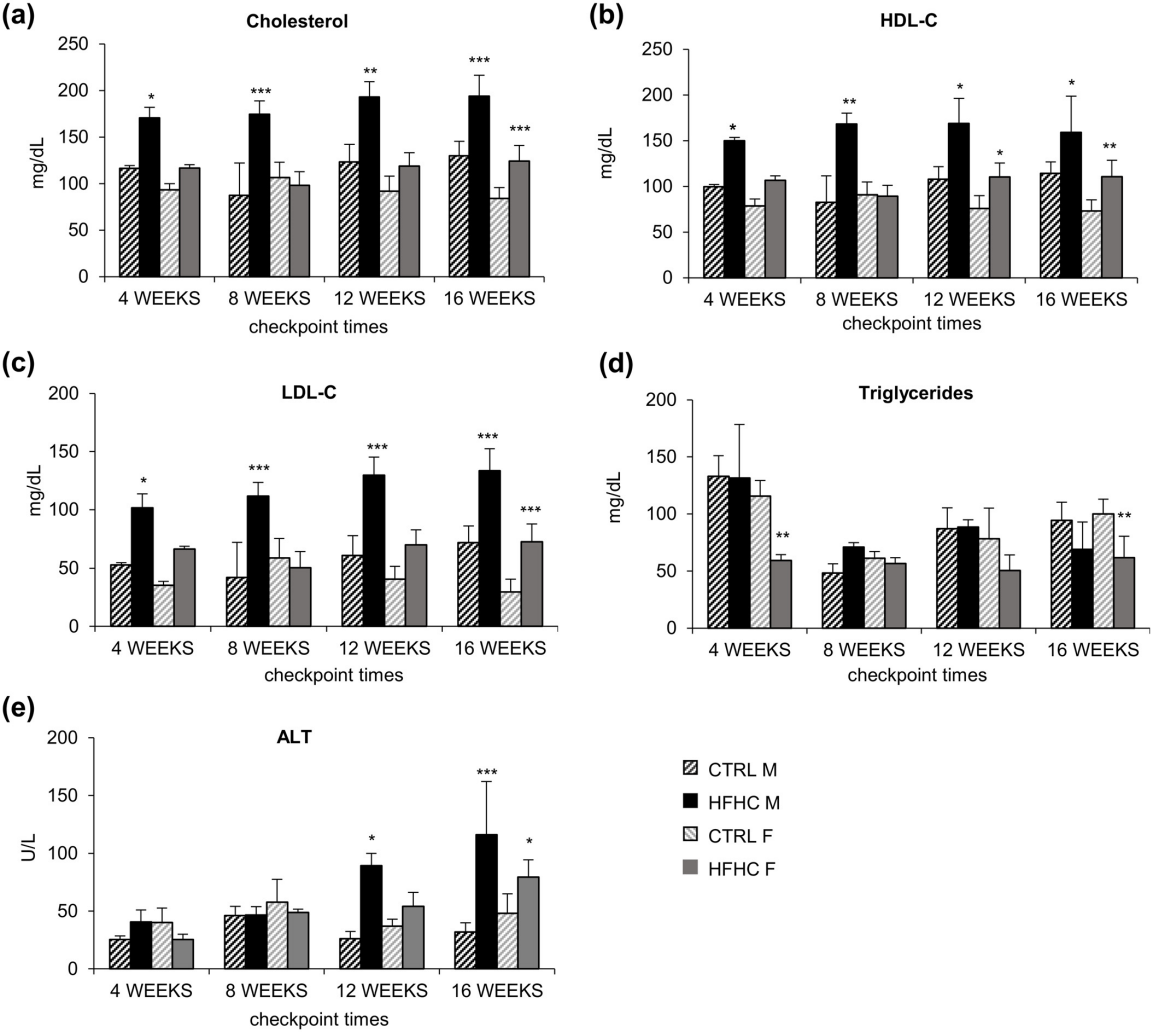
Lipid profile analysis and serum ALT. **(a)** Total cholesterol. **(b)** HDL-C. **(c)** LDL-C. **(d)** Triglycerides. **(e)** ALT. Data are expressed as mean ± SD. Statistical significance was calculated *vs*. age and sex matched CTRL. * p<0.05, ** p<0.01, *** p<0.001.

Also the lipid profile showed a behavior different in HFHC males and females. In males, total cholesterol (Chl), HDL-C and LDL-C levels (Fig 3a, 3b and 3c) rose steadily since week 4 (p<0.05) to week 16 (Chl and LDL-C, p<0.001 and HDL-C p<0.05), whereas in females a significant variation was observed only at week 16 (Chl and LDL-C, p<0.001 and HDL-C p<0.01). Triglycerides level was unchanged in males and decreased in females at all experimental times (even if the statistical significance was reached only at 4 and 16 weeks) (Fig 3d).

### Quantification of aminotransferases

Abnormal levels of serum ALT activity were found in HFHC males from week 12 (p<0.05, Fig 3e), increasing thereafter (16weeks: p<0.001). On the contrary, females had a peak in ALT at week 16 (p<0.05) (Fig 3e).

### Histopathological analysis

**Fig 4:**
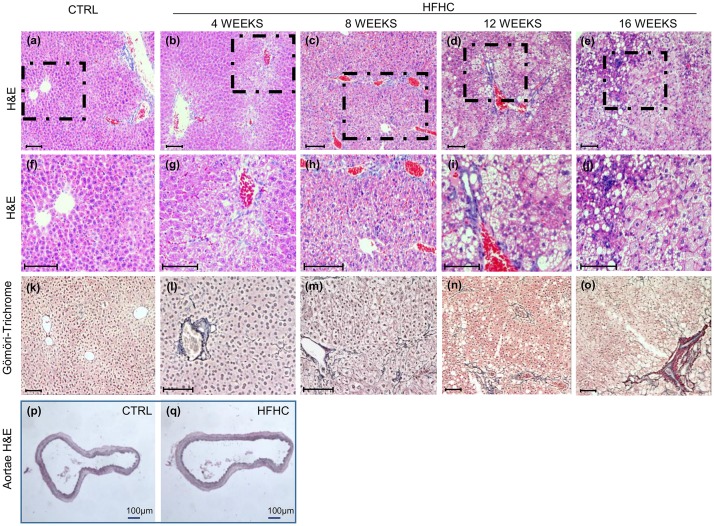
Hepatic and aortae histological findings. Hepatic histopathological analysis. **(b-e)** Representative images of Hematoxylin-Eosin staining progression of HFHC group over the time *vs*. CTRL **(a).** Higher magnification of the dashed area **(f-j). (l-o)** Gömöri trichrome staining for collagen of HFHC group over the time *vs*. CTRL **(k)**. **(p, q)** Representative picture of Hematoxylin & Eosin staining in aortae of controls **(p)** and HFHC group **(q).** In all pictures scale bar is 100 μm.

Hepatic steatosis (grade and location), fibrosis (grade and location), portal and lobular inflammation and ballooning were assessed according to Brunt’s classification [33]. Localization, score and percentage of animal showing abnormalities are detailed in Table 2. HFHC diet induced a macro-microvesicular steatosis in the 100% of males at week 4, progressing to more severe grades along the experimental period (Fig 4a–4o; Table 2). Similarly, score 2 steatosis was detected in all female mice at 4 and 8 weeks (Table 2, right side) reaching score 3 at 12 weeks.

**Table 2:** Histopathological findings. Histological analysis of the liver at each experimental checkpoint times according to Brunt's classification. Data is expressed as score/grade and between brackets the occurrence.

	MALE				FEMALE			
Parameters	4 weeks	8 weeks	12 weeks	16 weeks	4 weeks	8 weeks	12 weeks	16 weeks
Steatosis Grade (0–3)	1 (33%); 2(66%)	2 (100%)	2 (100%)	2 (33%); 3 (66%)	2 (100%)	2 (100%)	2 (33%); 3 (66%)	1 (29%); 2 (71%)
Location (score 0–3)	2; Azonal	2; Azonal	2; Azonal	3; Paracinar	2; Azonal	2; Azonal	2 (33%); Azonal. 3 (66%); Paracinar	1 (29%); periportal. 2 (71%); Azonal
Portal Inflammation (score 0–1)	0 (100%)	0 (66%); 1 (33%)	0 (100%)	0 (62%); 1 (28%)	0 (66%); 1 (33%)	0 (66%); 1 (33%)	0 (66%); 1 (33%)	0 (58%); 1 (42%)
Lobular Inflammation Score (0–3)	0 (66%); 1 (33%)	0 (33%); 1 (33%); 2 (33%)	0 (33%); 1 (66%)	0 (15%); 1 (85%)	0 (33%); 1 (66%)	0 (33%); 1 (66%)	0 (33%); 1 (66%)	0 (72%); 1 (28%)
Fibrosis Stage (0–4)	0 (66%); 1a (33%)	1b (66%); 1c (33%)	1a (33%); 1c (66%)	1a (14%); 2 (86%)	0 (66%); 1a (33%)	1a (66%); 1b (33%)	1a (33%); 1b (33%); 2 (33%)	1b (14%); 1c (71%); 2 (15%)

Lobular inflammation was observed in males starting from week 4 and increased along the experimental time. Increasing portal fibrosis was present in both males and females mice at week 4, reaching 100% of animals at week 8. At week 16, perisinusoidal and portal/periportal fibrosis (stage 2) was present in the 86% of males and 15% of females while ballooning injury was rare. Mallory-Denk bodies were not found at any experimental checkpoint. No signs of any morphological/atherogenic changes were observed in every sample of aorta studied (Fig 4p and 4q).

### Biomolecular analysis of the fibrotic processes activation

**Fig 5:**
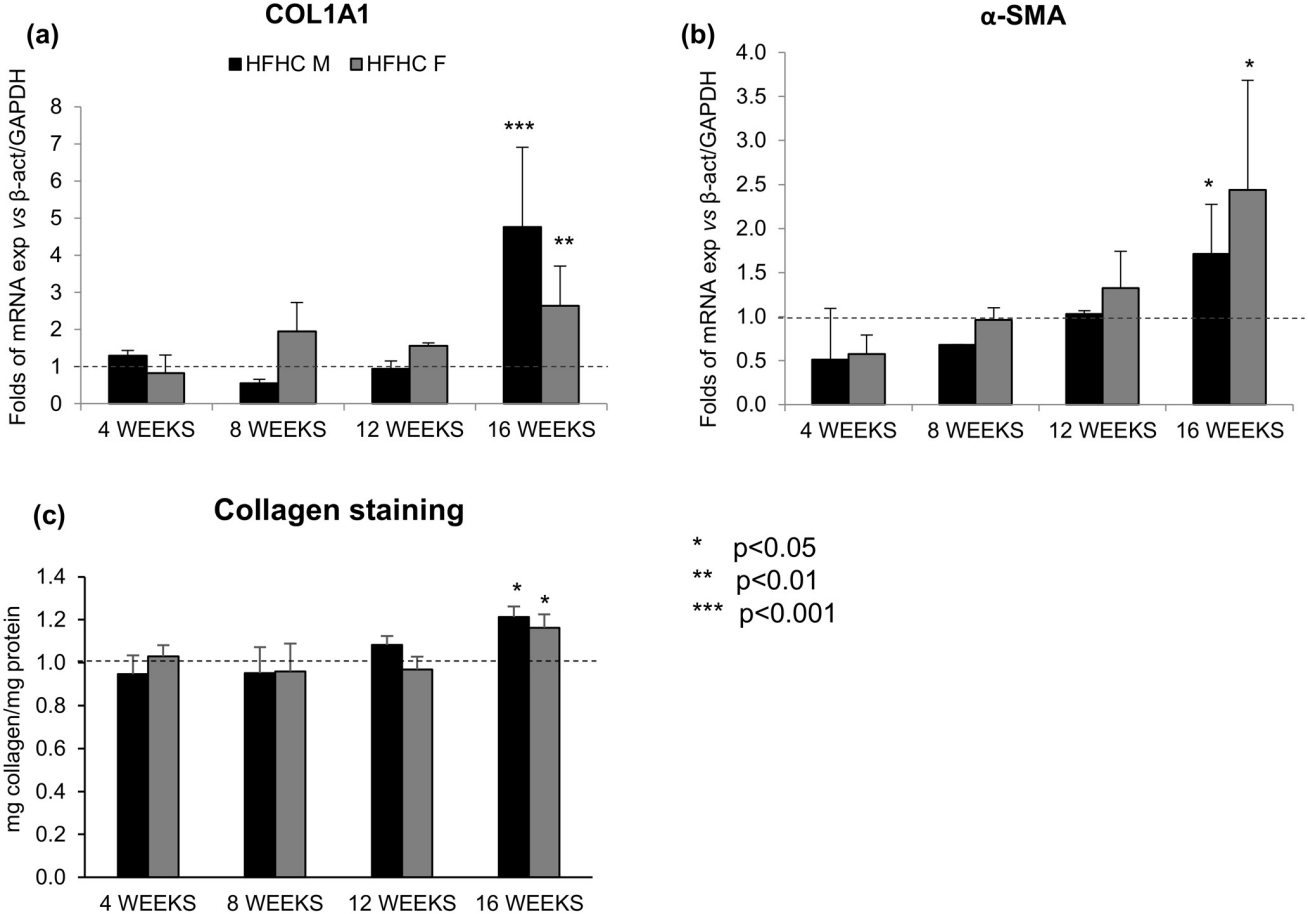
Real Time PCR and Sirius red-Fast Green staining. **(a)** mRNA expression of Col1A1 and **(b)** α-SMA. **(c)** Collagen protein quantification.

**Fig 6:**
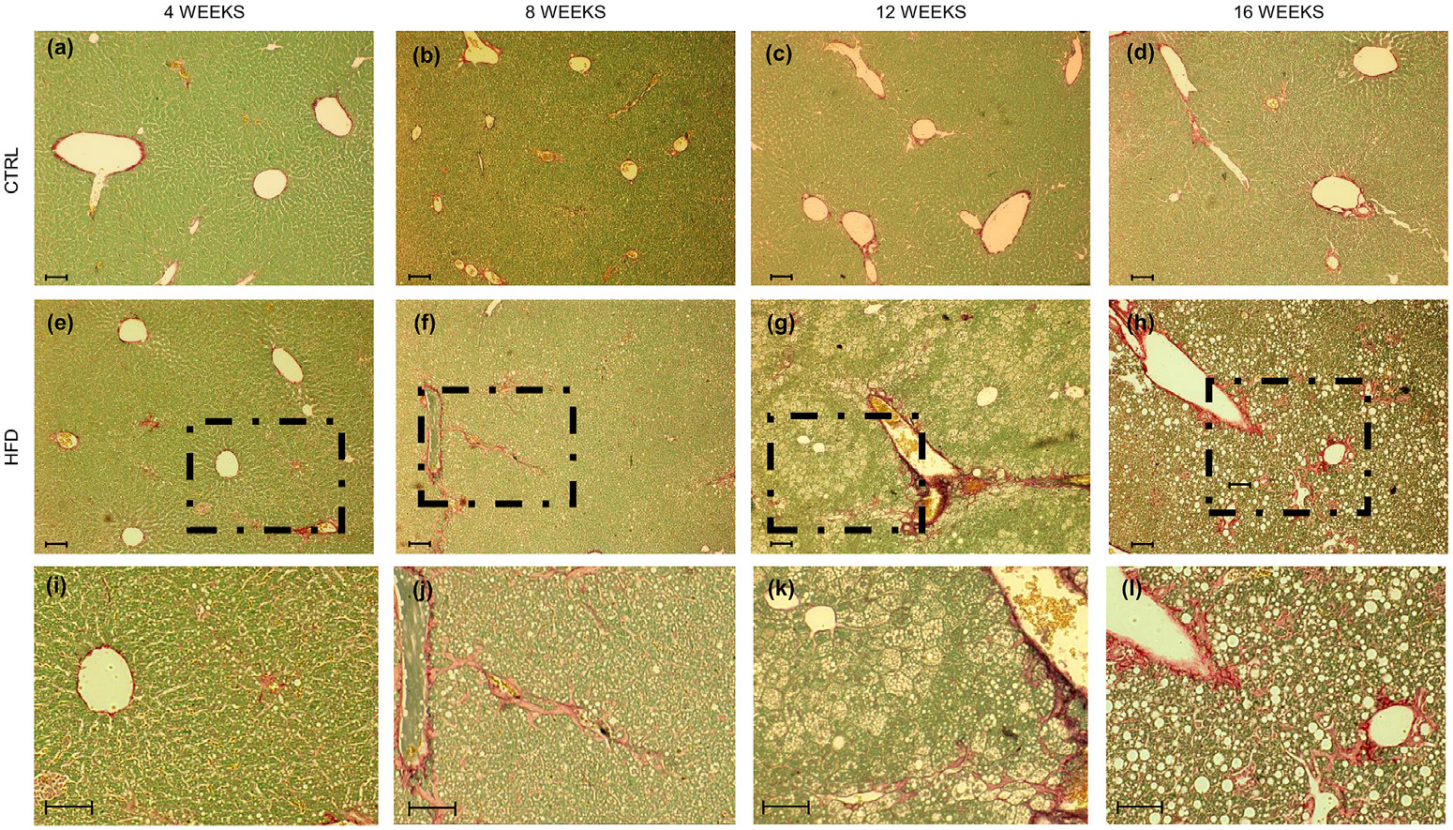
Hepatic histopathological analysis of extracellular matrix. Representative images of Sirius Red staining. In red collagen fibers, in green non-collagenous protein. **(a-d)** Controls and **(e-h)** HFHC animals o**v**er the time. **(i-l)** Higher magnification of the dashed area. In all pictures scale bar is 250 μm.

To confirm the presence of fibrosis, the modulation of Col1A1 and α-SMA (Fig 5a and 5b) was followed over the time, showing a statistical increase in both genders (Col1A1: p<0.001 and p<0.01 in males and females, respectively; α-SMA p<0.05, both sexes). Furthermore, Sirius Red/ Fast Green staining showed a perisinusoidal towards portal/periportal collagen positivity over the time (Figs 5c and 6a–6l).

Indeed the mRNA expression of Col1A1 was significantly up-regulated in both males and females (p<0.05 for both). Similarly, the gene expression of α-SMA was significantly increased in all the HFHC mice (p<0.05) (Fig 5a and 5b).

### Oxidative stress determination by MDA quantification

To assess the hepatic oxidative stress, another possible trigger for hepatic fibrosis, the amount of MDA was determined at every check point time. A significant increase in the MDA content (3.86 ± 0.37-folds, p<0.001) was observed only in females at 16 weeks of HFHC diet whereas males never showed differences.

## Discussion

Over the past years have been proposed several models to study the metabolic syndrome and the associated hepatic manifestation (NAFLD/NASH). These models are fundamental in the mechanistic studies of the onset and progression of the pathology. To be useful, a model has to mimic as closely as possible the human condition, from the causative (etiology) to the final biological, biochemical and histological clinical features. Nevertheless and despite the large literature, a remarkable gap exists in the availability of models devoted to the adolescent age, in spite of the booming of juvenile obesity and of its hepatic implication among adolescents and children.

In addition to the mutant mice and deficient diet models (presenting several limitations and pitfall in reproducing the human condition) a plethora of hypercaloric diets (differing in the total energy count, fat amount and lipid origin -vegetal, milk, lard or directly cholesterol) have been used [18–21,34]. Often, these differences have been made on purpose to investigate the consequences and the biomolecular mechanisms elicited by specific components present on the diet on the development of diabetes, atherosclerosis or different degrees of liver damage, goal that usually is well accomplished [18,20,35,36].

Our aim was to develop and characterize the first consistent juvenile model of NAFLD/NASH considering both sexes. We chose one of the most promising diets among all the existing, characterized by high caloric intake mainly provided by fats, plus fructose added in drinking water [25]. Fructose addition was reported to be associated with liver fibrosis (stage 2) [18–21] and moreover it mimics the increased consumption of high-fructose corn syrup in human alimentary habits [21].

Mice pups of 3weeks old (corresponding to a 3–3.5 years old child) fed with the HFHC, early showed the features of metabolic syndrome observed in humans which was associated with the histological pattern of the juvenile NAFLD/NASH [1,37].

Notably, already after 4weeks of HFHC diet, 100% of mice presented a mixed macro-microvesicular steatosis, while fibrosis (1a-1c) was present in all the animals after 8week of diet. This temporal pattern is much faster than what reported in the comparable adult models which require at least 3-4weeks for a significant body weight gain, 6–10 weeks for plasma lipid alteration [22,25,38] and more than 6weeks for the first recognition of histological changes [26].

Even more important is the observation that in our juvenile model the percentage of animals with relevant hepatic fibrosis (stage 2) at 16 weeks was higher than adult mice [25]. This faster progression to more severe stages might reproduces what suggested in the clinical scenario by the retrospective study of Feldstein [6].

The reason why the progression of liver damage is faster in juvenile that adult animals is still undefined. The hormonal and metabolic changes taking place in puberty could have a role, as suggested for humans [1,4,5]. During puberty, adiponectin level decreases as well as insulin sensitivity. On the contrary, sex hormones increase, with a redistribution of the body fat. Mice puberty occurs in 6wks-old females and 8wks old males [39] and this was the reason why we decide to start the HFHC diet immediately after weaning. An age dependent effect was previously reported in rats exposed to high fat vs. high fructose diets [40], demonstrating that the fat diet was more noxious than fructose in 4weeks old animals, and the opposite in 12weeks old rats. This study did not evaluate the combination of both fat and fructose. A similar age-dependent response to stimulus (cholesterol containing diet) was described in rabbits, with younger animals presenting lower serum HDL cholesterol (23%) and larger atherosclerotic areas (32%) than adults [41].

The developmental passage from childhood to adulthood has been involved also to explain the passage from the pediatric (the so called type 2: portal inflammation/fibrosis) to the adult (type 1: perisinusoidal) pattern of hepatic histological lesions [1,42,43]. In our model, a mixed type 1, type 2 inflammation was observed, with the lobular component increasing in males over the time. This may reflect the overlapping pattern observed in clinical studies with mixed features in 30–52% biopsies in adolescents with frank NASH [1,4,37,44,45]. This mixed pattern of hepatic lesions, as well a mico-macrovesicular steatosis have been observed also in a NASH model obtained by exposing 4-6week old male rabbits to a diet rich in fat by lard (10%) and cholesterol (2%) [46]. Also in this juvenile model which unfortunately did not include females, fibrosis reached stage 2.

Another difference with previously published works is the fact that we studied both males and females. In clinic, the prevalence of NAFLD/NASH is higher in males, increasing in females after menopause [10,11], with accumulation of visceral fat [12] due to the estrogen decline [13]. A liver protective role of estrogens has been described in animal models (e.g.: [22]) in which estrogens seem to inhibit both the gain in body weight, the increase of central adiposity and the pro-inflammatory status [1,22–24], possibly inhibiting the insulin-resistance [22].

In our juvenile model, we observed a comparable hepatic damage in males and females after 16 weeks of HFHC diet in spite of a sex related different time course of the alterations and the absence of insulin resistance in females.

This observation suggests that the underlying mechanisms in our juvenile model differ in the two sexes with a progressive portal and lobular inflammation in absence of lipid-peroxidation products in males, and the opposite in females. This is in line with data obtained in adult diet-induced models [47,48], in which it was reported that females (but not males) had an increased ability to contrast the pro-inflammatory status usually associated with obesity [22]. Interestingly, female mice never developed insulin resistance either in our juvenile or in the adult models [47].

## Conclusions

In summary the rapid evolution, the macroscopical and histological features we described in our mouse model well reproduced the differences in the pathogenesis of NAFLD/NASH reported between children and adults. Our data indicate that the development of fibrosis may be dictated by different mechanisms between males and females, with a possible masking effect of hormones in the latter. Our model fills a relevant gap in the study of this juvenile liver injury progression and may provide a suitable platform for the pre-clinical evaluation of focused treatments for NAFLD/NASH in both sexes.
